# Palovarotene for patients with multiple hereditary exostosis: results of MO-Ped, a terminated, randomized, placebo-controlled, double-blind phase 2 trial

**DOI:** 10.1038/s41598-025-22554-6

**Published:** 2025-11-04

**Authors:** Luca Sangiorgi, Ernest U. Conrad, Fei Shih, Andrew Strahs, David S. Feldman

**Affiliations:** 1 Medical Genetics and Skeletal Rare Diseases, IRCCS Istituto Ortopedico Rizzoli, Bologna, Italy; 2 Department of Orthopedic Surgery, University of Texas Health Science Center, Houston, TX USA; 3 Ipsen, Cambridge, MA USA; 4 Paley Advanced Limb Lengthening Institute, St. Mary’s Hospital, West Palm Beach, FL USA

**Keywords:** Clinical trial, Multiple hereditary exostosis, Osteochondromas, Palovarotene, Premature physeal closure, Diseases, Medical research, Mutation

## Abstract

A phase II trial (MO-Ped; NCT03442985) assessed palovarotene in pediatric patients with multiple hereditary exostosis (MHE). Patients aged ≤ 14 years with MHE were randomized 1:1:1 to placebo, palovarotene 2.5 mg, or palovarotene 5.0 mg daily. Due to concerns of premature physeal closure (PPC), a partial clinical hold was instituted, followed by trial termination. The primary efficacy endpoint was the annualized rate of new osteochondromas (OCs). Safety was assessed. Overall, 193 patients received ≥ 1 dose of palovarotene or placebo. Due to trial termination, no patients completed planned treatment. Prior to 06 December 2019 (patients notified of clinical hold and treatment stopped), 30 patients completed Month 12 efficacy imaging. No significant differences in the annualized rate of new OCs, or change from baseline in volume of OCs or OC cartilage, were observed between treatment groups. The adverse event profile was consistent with systemic retinoids. There was no evidence of an effect of palovarotene on linear growth and no cases of PPC. Overall, palovarotene showed no clear efficacy signal in MHE, resulting in a non-favorable benefit-risk profile. Interpretation of results was limited by the reduced treatment duration and smaller than expected cohort. The trial yielded important data on the natural history of MHE.

*Trial registration*: NCT03442985 (first posted 22 February 2018)

**Supplementary Information:**

The online version contains supplementary material available at 10.1038/s41598-025-22554-6.

## Introduction

Multiple hereditary exostosis (MHE), also known as multiple osteochondromas (OCs), is an autosomal-dominant genetic disorder that, although rare, is among the most common inherited musculoskeletal disorders^1–3^. The majority (~ 90%) of MHE cases are associated with heterozygous loss-of-function variants of exostosin 1 (*EXT1*) or exostosin 2 (*EXT2*), which encode an essential glycosyltransferase for heparan sulfate biosynthesis^3–6^. MHE is characterized by the growth of multiple cartilage-capped bone tumors, known as OCs, which arise from the periphery of growth plates situated in long bones or the surface of flat bones^5,7,8^.

Generally, the development of OCs is limited to the first two decades of life^1,3^. OCs grow in size and gradually ossify during skeletal development, and usually stop growing once skeletal maturity is reached^9^. Although OCs can be asymptomatic, signs and symptoms include limb deformities, impaired range of motion, and dislocation of joints^3,8,10^. Pain can also develop from the compression of adjacent structures, such as nerves, tendons, and muscles^3,10^. The number, size, and location of OCs, and prevalence of complications can vary substantially among patients^10,11^. Malignant transformation of OCs occurs in approximately 0.5–10% of MHE cases^3,12,13^.

Currently, there are no treatments available to prevent OC development in MHE^3^. In cases where OCs cause pain, compromise joint movement, or impinge adjacent structures, surgical excisions can be performed; however, patients may be at risk of surgery-related pain or further complications^14,15^. Palovarotene is an orally bioavailable, selective retinoic acid receptor (RAR)-γ agonist that has been approved for the treatment of fibrodysplasia ossificans progressiva (FOP)^3,16–18^. The pharmacokinetic and pharmacodynamic profile of palovarotene following oral administration has been well-characterized, and findings support daily administration^19,20^. Research in mouse models has shown that palovarotene could be effective in preventing OC growth in patients with MHE^4^.

Results are presented from MO-Ped, a phase II trial which assessed efficacy and safety of palovarotene in pediatric patients with MHE^21^. A plain language summary of this trial is provided in the Supplementary Information.

## Methods

### Trial design

MO-Ped (PVO-2A-201; NCT03442985; first posted 22 February 2018) was a phase II, randomized, placebo-controlled, double-blind trial designed with three periods: an initial screening period (up to 35 days in duration), a 24-month double-blind treatment period, and a 6-month safety follow-up period (Supplementary Fig. 1). Patients eligible to participate were aged 2–14 years with a clinical diagnosis of symptomatic MHE resulting from a *EXT1* or *EXT2* pathogenic variant. Patients were randomized 1:1:1 to receive daily placebo, palovarotene 2.5 mg, or palovarotene 5.0 mg using a centralized interactive web response system. Randomization was stratified by age (≤ 7 years and > 7 years), sex, and disease-causing gene variant. Additional details on trial design are provided in the Supplementary Information and Supplementary Table 1. The trial was conducted in accordance with the ethical principles outlined in the Declaration of Helsinki; the protocol was reviewed and approved by independent review boards/independent ethics committees local to each site prior to study initiation (details for each site are provided in Supplementary Table 2).

### Partial clinical hold and trial termination

Due to concerns of premature physeal closure (PPC) in studies of palovarotene in FOP^18^, a partial clinical hold was instituted by the U.S. Food and Drug Administration (FDA) for patients aged < 14 years on 04 December 2019, whereby all recruitment and dosing in MO-Ped was stopped^22^. Patients were notified on 06 December 2019 and were asked to remain in the trial and complete their scheduled visits. On 24 March 2020, the trial was terminated, with patients encouraged to return for a safety follow-up visit 6 months after the end of treatment.

### Efficacy outcomes

The schedule of assessments is shown in Supplementary Table 3. The primary efficacy endpoint was the annualized rate of new OCs. Secondary efficacy endpoints included change from baseline in volume of OCs and change from baseline in volume of OC cartilage caps. Primary and secondary efficacy endpoints were assessed by whole-body magnetic resonance imaging (MRI); details on MRI acquisition and analysis are provided in the Supplementary Information.

Additional efficacy endpoints included: proportion of patients with no new OCs as assessed by whole-body MRI; annualized rate of new or worsening skeletal deformities or functional limitations as assessed by radiographs of the upper and lower limbs and clinical evaluations; and annualized rate of MHE-related surgeries. Due to trial termination, pre-planned efficacy analyses on patient-reported outcomes were not performed.

### Safety outcomes

Safety was monitored throughout screening, treatment, and follow-up periods. Comprehensive physical examinations were performed by physicians at clinic visits (Supplementary Table 3). Investigators were required to record any post-baseline abnormal findings as adverse events (AEs) and follow up on all AEs to the end of the reporting period or until symptoms stabilized. AEs were coded using the Medical Dictionary for Regulatory Activities (MedDRA; version 22.0). Additional detail on AE definitions, and bone and other safety outcomes are provided in the Supplementary Information.

### Sample size

The sample size required was determined by simulation using a 1-sided, overall type I error rate of 1.25% for each palovarotene group comparison with placebo. OC counts were assumed to follow a negative binomial distribution parameterized with a variance inflation factor of 2.0. The annualized rate of new OCs was assumed as 1.15, based on analyses performed by the Istituto Ortopedico Rizzoli (IOR) Registry for Multiple Exostoses (REM) at the time the protocol was developed^23^.

### Statistical analyses

**Fig. 1:**
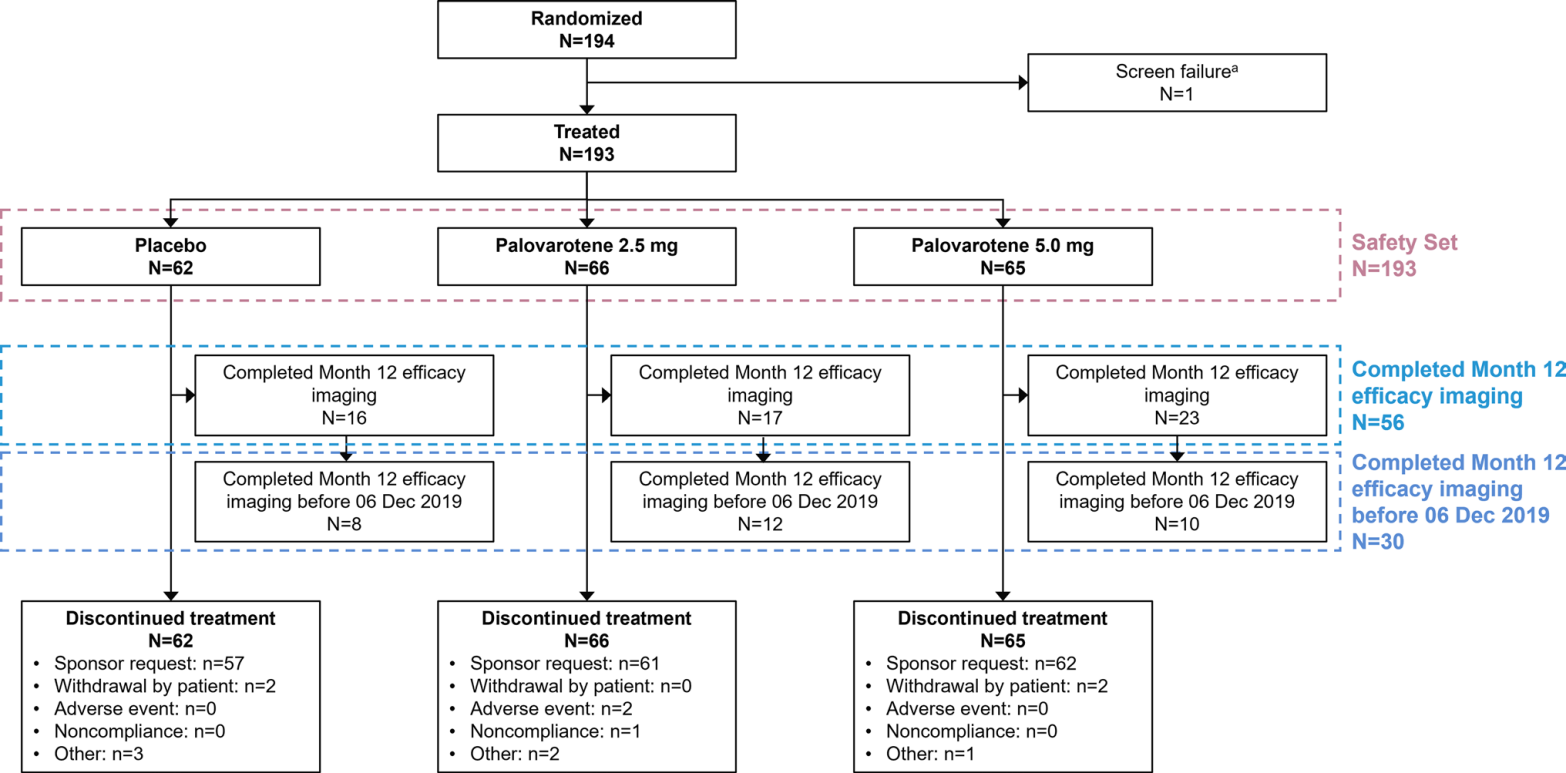
Patient disposition. ^a^One individual who failed screening for the trial was mistakenly randomized to placebo before being withdrawn prior to treatment.

The Full Analysis Set and Safety Set included randomized patients who received ≥ 1 dose of study treatment (Fig. 1). To account for variability in treatment duration, efficacy analyses were conducted for patients in the Full Analysis Set who: (1) completed Month 12 efficacy imaging before study treatment discontinuation on 06 December 2019 (when treatment permanently stopped), or (2) completed Month 12 efficacy imaging at any time (before or after 06 December 2019; Fig. 1). Safety analyses used the Safety Set. Bone mineral content (BMC) and areal bone mineral density (aBMD) were also reported in patients who completed Month 12 efficacy imaging at any time. All analyses were summarized by treatment group (placebo, palovarotene 2.5 mg, and palovarotene 5.0 mg), including pooled palovarotene-treated patients for select outcomes. Additional detail on statistical analyses are provided in the Supplementary Information.

## Results

### Patient disposition

In total, 194 patients were randomized between March 2018 and December 2019, with 193 patients having received ≥ 1 dose of palovarotene or placebo (Safety Set: placebo, *N* = 62; palovarotene 2.5 mg, *N* = 66; palovarotene 5.0 mg, *N* = 65; Fig. 1).

Due to early trial termination, no patients completed the planned treatment schedule (maximum treatment duration: 595 days), resulting in a considerably underpowered study for hypothesis testing. Month 12 efficacy imaging was completed by 56 patients, of which 30 completed this before palovarotene dosing was stopped (06 December 2019; Fig. 1). The 30 patients who completed Month 12 efficacy imaging before 06 December 2019 received ≥ 1 year of treatment. The additional 26 patients who completed Month 12 efficacy imaging after 06 December 2019 received < 1 year of treatment. Efficacy analyses were not performed in the remaining randomized patients due to a lack of post-baseline efficacy imaging and limited treatment length.

### Baseline demographics and characteristics

Baseline demographics and characteristics for patients who completed Month 12 efficacy imaging before 06 December 2019 (placebo, *N* = 8; palovarotene 2.5 mg, *N* = 12; palovarotene 5.0 mg, *N* = 10) are described in Table 1. Demographics and characteristics were generally similar among treatment groups. Baseline demographics and characteristics for patients in the Safety Set and those who completed Month 12 efficacy imaging at any time are presented in Supplementary Tables 4 and 5, respectively.

**Table 1:** Baseline demographics and characteristics for patients who completed month 12 efficacy imaging prior to 06 December 2019.

	Placebo (*N* = 8)	Palovarotene 2.5 mg (*N* = 12)	Palovarotene 5.0 mg (*N* = 10)	All palovarotene-treated patients (*N* = 22)	All patients (*N* = 30)
**Sex, n (%)**					
Male	4 (50.0)	7 (58.3)	5 (50.0)	12 (54.5)	16 (53.3)
**Race**, **n (%)**					
White Asian Multiple	7 (87.5) 1 (12.5) 0	11 (91.7) 0 1 (8.3)	9 (90.0) 1 (10.0) 0	20 (90.9) 1 (4.5) 1 (4.5)	27 (90.0) 2 (6.7) 1 (3.3)
**Ethnicity**,** n (%)**					
Hispanic or Latino	0	2 (16.7)	0	2 (9.1)	2 (6.7)
**Age**,** years**					
Mean (SD)	6.4 (2.8)	6.8 (4.0)	8.1 (2.8)	7.4 (3.5)	7.1 (3.3)
Median (min, max)	6.0 (3, 11)	6.5 (2, 12)	8.5 (4, 11)	7.0 (2, 12)	7.0 (2, 12)
**Age group**,** n (%)**					
2–5 years	4 (50.0)	5 (41.7)	3 (30.0)	8 (36.4)	12 (40.0)
6–10 years	3 (37.5)	3 (25.0)	4 (40.0)	7 (31.8)	10 (33.3)
11–14 years	1 (12.5)	4 (33.3)	3 (30.0)	7 (31.8)	8 (26.7)
**MHE-associated variant**,** n (%)**					
*EXT1*	7 (87.5)	8 (66.7)	8 (80.0)	16 (72.7)	23 (76.7)
*EXT2*	1 (12.5)	4 (33.3)	2 (20.0)	6 (27.3)	7 (23.3)
**MHE-associated mutation type**,** n (%)**					
Missense	2 (25.0)	1 (8.3)	1 (10.0)	2 (9.1)	4 (13.3)
Nonsense	1 (12.5)	1 (8.3)	4 (40.0)	5 (22.7)	6 (20.0)
Silent	0	0	1 (10.0)	1 (4.5)	1 (3.3)
Donor-acceptor splice site	0	2 (16.7)	0	2 (9.1)	2 (6.7)
Copy number variants (exon-level deletions/duplications) and whole gene deletions/duplications	2 (25.0)	2 (16.7)	1 (10.0)	3 (13.6)	5 (16.7)
Intronic	0	0	1 (10.0)	1 (4.5)	1 (3.3)
Frame shift	3 (37.5)	6 (50.0)	2 (20.0)	8 (36.4)	11 (36.7)

*EXT1*, exostosin 1; *EXT2*, exostosin 2; max, maximum; MHE, multiple hereditary exostosis; min, minimum; SD, standard deviation.

### Efficacy

**Fig. 2:**
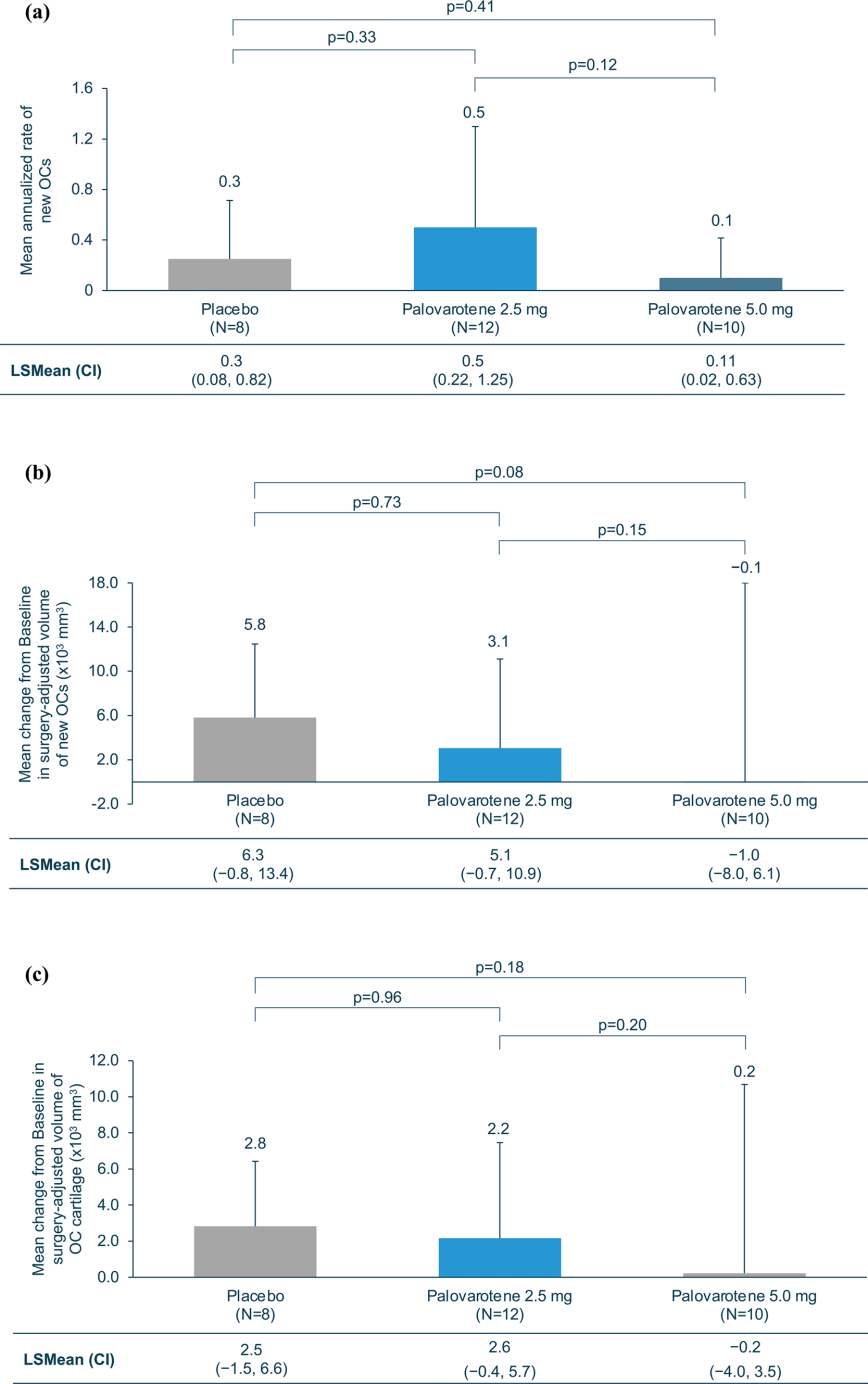
Efficacy results for patients who completed Month 12 efficacy imaging prior to 06 December 2019. (**a**) Annualized rate of new OCs at Month 12^a^. (**b**) Mean change from baseline in surgery-adjusted volume of OCs at Month 12. (**c**) Mean change from baseline in surgery-adjusted volume of OC cartilage at Month 12. ^a^Determined by the number of new OCs identified by MRI; therefore, surgically-removed OCs were not included. Error bars show SDs of the mean. CI, confidence interval; LSMean, least squares mean; MRI, magnetic resonance imaging; OC, osteochondroma; SD: standard deviation

In patients who completed Month 12 efficacy imaging before 06 December 2019 (*N* = 30), and therefore received ≥ 1 year of treatment, the observed number of new OCs, identified by MRI and excluding OCs removed by surgery, was low. Two new OCs were observed in eight patients who received placebo, six new OCs in 12 patients who received palovarotene 2.5 mg, and one new OC in 10 patients who received palovarotene 5.0 mg. No statistically significant differences in the annualized rate of new OCs were observed (Fig. 2a). Change from baseline in volume of OCs was similar between patients treated with placebo and palovarotene 2.5 mg; however, a non-significant decrease was observed in patients treated with palovarotene 5.0 mg (Fig. 2b). There was no significant change from baseline in volume of OC cartilage between patients treated with placebo and palovarotene 2.5 mg or palovarotene 5.0 mg (Fig. 2c).

Efficacy results for patients who completed Month 12 efficacy imaging at any time (*N* = 56) also showed a low observed number of new OCs (excluding those surgically removed prior to imaging). Two new OCs were observed in 16 patients who received placebo, six new OCs in 17 patients who received palovarotene 2.5 mg, and four new OCs in 23 patients who received palovarotene 5.0 mg. No statistically significant differences were observed between treatment groups in the annualized rate of new OCs or in change from baseline in volume of OCs or OC cartilage (Supplementary Fig. 2). When considering the total number of new OCs, including those removed by surgery, similar percentages of patients with no new OCs were observed in each treatment group: 62.5% (10/16) who received placebo, 47.1% (8/17) who received palovarotene 2.5 mg, and 56.5% (13/23) who received palovarotene 5.0 mg (Supplementary Fig. 3). The mean annualized rate of new or worsening deformities and rates of MHE-related surgeries was similar between treatment groups (Supplementary Fig. 3).

### Safety

Mean duration of exposure in the Safety Set (*N* = 193) for palovarotene was 218.0 days (range: 10–595 days; *n* = 130) and for placebo was 212.8 days (range: 10–561 days; *n* = 62). Dose modifications due to AEs were reported in 13.0% of patients treated with palovarotene and 4.8% with placebo.

Most patients reported ≥ 1 treatment-emergent AE (TEAE), with more TEAEs reported in palovarotene-treated patients compared with placebo (Table 2). Most TEAEs were assessed as mild. Of the most common TEAEs, higher frequencies were observed in palovarotene-treated patients, with the exceptions of arthralgia and headache, which were more frequent in the placebo-treated group (Table 3). The most common TEAEs observed in palovarotene-treated patients were mucocutaneous AEs.

**Table 2:** Overview of adverse events (Safety Set^a^).

	Placebo (*N* = 62)	Palovarotene 2.5 mg (*N* = 66)	Palovarotene 5.0 mg (*N* = 65)	All palovarotene-treated patients (*N* = 131)	All patients (*N* = 193)
**TEAE category, n (%)**					
Any TEAE	41 (66.1)	56 (84.8)	56 (86.2)	112 (85.5)	153 (79.3)
Possibly related TEAE	29 (46.8)	49 (74.2)	54 (83.1)	103 (78.6)	132 (68.4)
Serious TEAE	0	2 (3.0)	2 (3.1)	4 (3.1)	4 (2.1)
Possibly related serious TEAE	0	0	1 (1.5)	1 (0.8)	1 (0.5)
TEAE leading to death	0	0	0	0	0
TEAE leading to withdrawal from trial	0	0	0	0	0
TEAE leading to discontinuation of study treatment	0	2 (3.0)	0	2 (1.5)	2 (1.0)
**Post-treatment AE category, n (%)**					
Any post-treatment AE	17 (27.4)	17 (25.8)	14 (21.5)	31 (23.7)	48 (24.9)
Post-treatment SAE	1 (1.6)	0	3 (4.6)	3 (2.3)	4 (2.1)

^a^All patients who received at least one dose of study treatment. TEAEs were any AE with an onset date from first study treatment intake to 7 days after last study treatment intake. Post-treatment AEs were any AE with an onset date from 7 days after last study treatment intake to study completion/discontinuation. AE, adverse event; SAE, serious adverse event; TEAE, treatment-emergent adverse event.

**Table 3:** Common treatment-emergent adverse events (Safety Set^a^).

	Placebo (*N* = 62)	Palovarotene 2.5 mg (*N* = 66)	Palovarotene 5.0 mg (*N* = 65)	All palovarotene-treated patients (*N* = 131)	All patients (*N* = 193)
**Patients with any TEAE**,** n (%)**	41 (66.1)	56 (84.8)	56 (86.2)	112 (85.5)	153 (79.3)
**Skin and subcutaneous tissue disorders**,** n (%)**	21 (33.9)	43 (65.2)	51 (78.5)	94 (71.8)	115 (59.6)
Rash	7 (11.3)	17 (25.8)	25 (38.5)	42 (32.1)	49 (25.4)
Dry skin	7 (11.3)	16 (24.2)	19 (29.2)	35 (26.7)	42 (21.8)
Pruritus	6 (9.7)	8 (12.1)	7 (10.8)	15 (11.5)	21 (10.9)
Rash generalized	2 (3.2)	3 (4.5)	5 (7.7)	8 (6.1)	10 (5.2)
**Gastrointestinal disorders**,** n (%)**	12 (19.4)	24 (36.4)	23 (35.4)	47 (35.9)	59 (30.6)
Lip dryness	3 (4.8)	6 (9.1)	11 (16.9)	17 (13.0)	20 (10.4)
Vomiting	2 (3.2)	3 (4.5)	7 (10.8)	10 (7.6)	12 (6.2)
Dry mouth	3 (4.8)	4 (6.1)	4 (6.2)	8 (6.1)	11 (5.7)
**Infections and infestations**,** n (%)**	14 (22.6)	20 (30.3)	20 (30.8)	40 (30.5)	54 (28.0)
Nasopharyngitis	3 (4.8)	3 (4.5)	5 (7.7)	8 (6.1)	11 (5.7)
**Musculoskeletal and connective tissue disorders**,** n (%)**	10 (16.1)	6 (9.1)	9 (13.8)	15 (11.5)	25 (13.0)
Arthralgia	7 (11.3)	3 (4.5)	1 (1.5)	4 (3.1)	11 (5.7)
**General disorders and administration site conditions**,** n (%)**	2 (3.2)	8 (12.1)	10 (15.4)	18 (13.7)	20 (10.4)
Pyrexia	0	3 (4.5)	9 (13.8)	12 (9.2)	12 (6.2)
**Nervous system disorders**,** n (%)**	8 (12.9)	5 (7.6)	6 (9.2)	11 (8.4)	19 (9.8)
Headache	6 (9.7)	4 (6.1)	3 (4.6)	7 (5.3)	13 (6.7)

^a^All patients who received at least one dose of study treatment. Common TEAEs were defined as having an incidence of ≥ 5% in any treatment group by system organ class and preferred term. TEAEs were any adverse event with an onset date from first study treatment intake to 7 days after last study treatment intake. AE, adverse event; TEAE, treatment-emergent adverse event.

Serious TEAEs were reported in two (3.0%) patients who received palovarotene 2.5 mg (radius and ulna fracture in one patient and pneumonia in one patient), and two (3.1%) patients who received palovarotene 5.0 mg (status epilepticus in one patient and blood loss anemia in one patient), whereas none were reported in patients who received placebo. One serious TEAE observed in the palovarotene 5.0 mg group was considered possibly related to study treatment (status epilepticus).

No patients experienced TEAEs leading to death or withdrawal from the trial. Two patients treated with palovarotene 2.5 mg had TEAEs leading to treatment discontinuation; one being a decreased appetite, assessed as moderate and possibly related to study treatment, and the other being an arthropod bite, assessed as moderate and not related to study treatment.

No cardiac or vital sign-related safety signals were observed and no patients reported Type 4 or Type 5 suicidal ideation.

### Bone safety

At baseline, patients with MHE tended to be of shorter stature than their normative peers; however, no significant differences in linear growth from baseline through Month 18 were observed between palovarotene- and placebo-treated patients. Additionally, changes from baseline in knee height did not reveal clinically relevant differences between patients who received placebo or palovarotene, and no patients experienced growth arrest.

Incidence of PPC was evaluated due to concerns reported in studies of palovarotene in FOP^18,22^. No patients were reported to have PPC. Partial or complete epiphyseal closures were identified in two patients who received placebo, three who received palovarotene 2.5 mg, and four who received palovarotene 5.0 mg. These patients were aged ≥ 11 years and epiphyseal closures were evaluated as appropriate with respect to their age and maturation status.

**Fig. 3:**
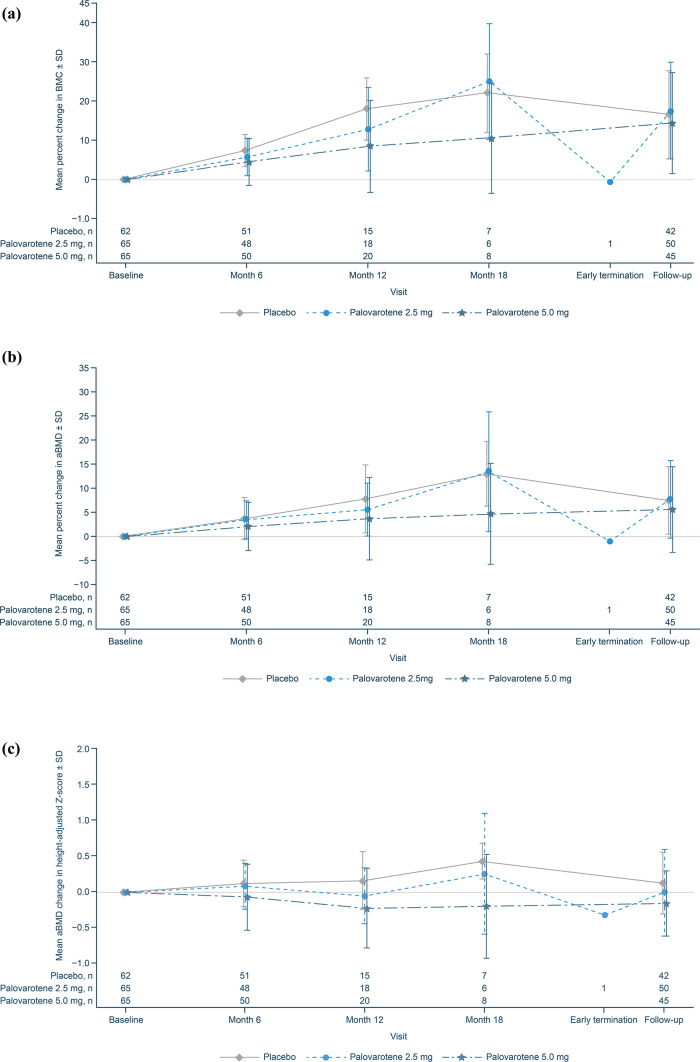
Change from Baseline in BMC and aBMD for patients in the Safety Set. (**a**) Percent change from baseline in BMC of the spine. (**b**) Percent change from baseline in aBMD of the spine. (**c**) Height-adjusted Z-score mean change from baseline in aBMD of the spine. Error bars show SDs of the mean. aBMD, areal bone mineral density; BMC, bone mineral content; SD, standard deviation.

In the Safety Set, there were no notable differences in BMC of the spine at baseline between patients who received placebo or palovarotene. Consistent with physiologic growth, BMC increased from baseline through Month 18; however, at Month 12, a dose-dependent reduction in BMC accrual of the spine was observed in palovarotene-treated patients. In the palovarotene 5.0 mg group, this trend persisted at Month 18, but returned to levels similar to placebo at the follow-up visit (Fig. 3a). Similar trends were observed for aBMD of the spine and aBMD using height-adjusted Z-scores (Fig. 3b and c).

**Fig. 4:**
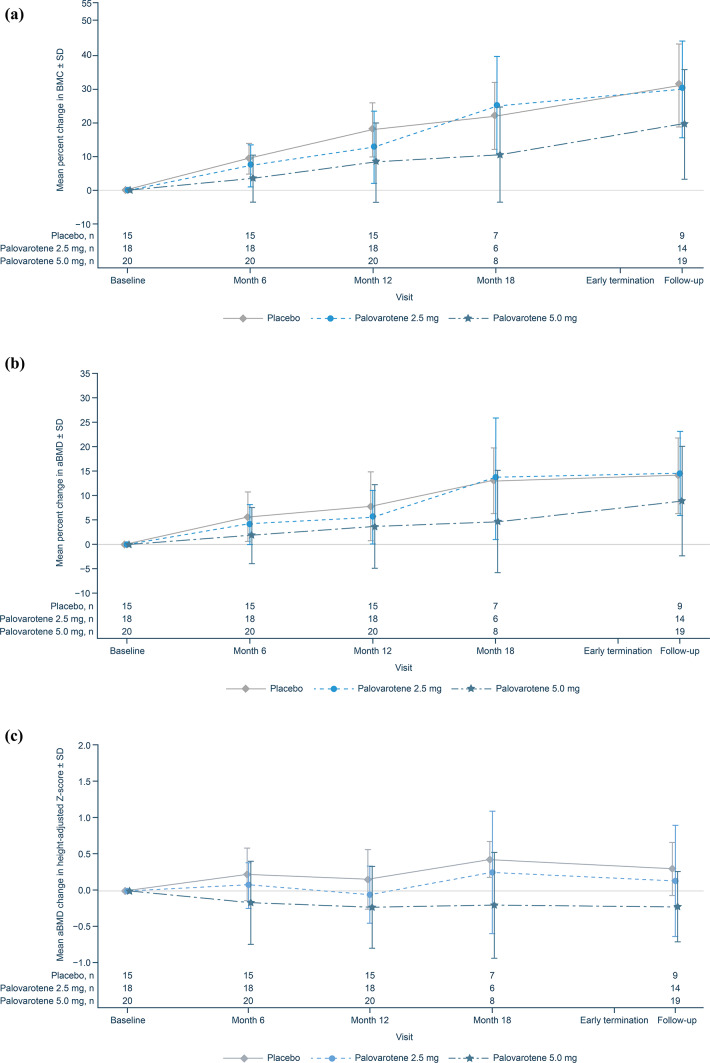
Change from baseline in BMC and aBMD for patients who completed Month 12 efficacy imaging. (**a**) Percent change from baseline in BMC of the spine. (**b**) Percent change from baseline in aBMD of the spine. (**c**) Height-adjusted Z-score mean change from baseline in aBMD of the spine. Error bars show SDs of the mean. aBMD, areal bone mineral density; BMC, bone mineral content; SD, standard deviation.

In all patients who completed Month 12 efficacy imaging, the attenuated accrual in BMC of the spine in the palovarotene 5.0 mg group persisted at the follow-up visit, which was not observed in the placebo or palovarotene 2.5 mg groups (Fig. 4a). This trend was also observed for aBMD of the spine and aBMD using height-adjusted Z-scores (Fig. 4b and c). Three patients who received palovarotene 5.0 mg had a ≥ 5% decrease in spine aBMD and a ≥ 1 point decrease in height-adjusted Z-score from baseline. For one patient, this was evaluated as a mild, non-serious TEAE of decreased bone density. For the other two patients, these changes were attributed to extenuating circumstances of surgeries and/or growth spurt by the Investigator and were not recorded as AEs.

Bone fractures occurred in two patients who received placebo and three patients who received palovarotene 2.5 mg, and were generally observed in the upper extremity and associated with trauma. No vertebral compression fractures were detected. Evaluation of osteonecrosis found abnormalities in two patients who received placebo, two who received palovarotene 2.5 mg, and five who received palovarotene 5.0 mg. One patient treated with palovarotene 5.0 mg presented with possible new onset osteonecrosis at Month 12; however, this was not clinically confirmed by the Investigator.

## Discussion

There are currently no effective treatments to prevent OC development in MHE^3^. Here, data were presented from MO-Ped, which was designed to evaluate the efficacy and safety of palovarotene in pediatric patients with MHE^21^. Trial recruitment and treatment was stopped prior to full enrollment because of early trial termination. As a result, a smaller than expected number of patients were enrolled, and patients were exposed to a shorter than expected duration of treatment. This lower sample size coupled with the unexpectedly low OC incidence rate observed in the placebo arm preclude demonstration of a statistical conclusion.

Overall, there was an absence of a clear efficacy signal in the prevention of new OCs or OC growth in patients who received palovarotene versus placebo. Treatment with palovarotene 5.0 mg was associated with a non-statistically significant reduction from baseline in OC and OC cartilage volume, which may be noteworthy since OC volumes rarely decline during the natural course of MHE. Spontaneous resolution of OCs has been reported^24,25^; however, to our knowledge, spontaneous resolution of OCs in MHE is rare, based on a lack of published literature. This being said, interpretation of the results is limited by the shorter than expected treatment duration, with 26 of the patients who completed Month 12 efficacy imaging having < 1 year of exposure, and the smaller than expected number of patients enrolled. In addition, > 70% of patients enrolled were aged ≤ 10 years, and therefore were less likely to develop new OCs because of their relative youth^26^, which reduced the statistical power of the analyses. Together, these limitations prevent definitive conclusions regarding efficacy.

Results from MO-Ped may improve our understanding of the natural history of MHE. The annualized rate of new OCs in patients aged 3–11 years who received placebo was 0.13, which was lower than the annualized rate of 0.35–0.60 in patients aged 0–12 years, reported in an Italian 36-month natural history study^26^. Mordenti et al. (2021) reported that patients with MHE aged < 10 years had milder disease symptoms compared with patients aged 10–15 and ≥ 16 years^27^. It is important to note that the assumed annualized rate of new OCs of 1.15, used to power the study, was later reassessed and lowered to 0.49 over 36 months by the IOR group, which is more consistent with these results^26^.

The AE profile of palovarotene was consistent with that of systemic retinoids^28,29^. Systemic retinoids are associated with effects on the musculoskeletal system, including PPC, osteoporosis, and increased fracture risk^30^, with cases of PPC previously observed in patients with FOP receiving palovarotene^18,22^.

No evidence of an effect of palovarotene on linear growth was observed in MO-Ped, and no cases of PPC were identified, with all post-baseline closures deemed physiologic, though this may be due to the short treatment duration. However, a trend of decreased BMC accrual and aBMD loss of the spine was observed in patients who received palovarotene 5.0 mg, which stabilized after treatment discontinuation. This stabilization in bone mass accrual could be attributed to a dilution in reported differences, since the follow-up visit included patients who received shorter periods of treatment, due to trial termination. Given the adverse bone safety effects observed with palovarotene here and in clinical trials of FOP^18^, and the known class effects of systemic retinoids^28,29^, bone safety monitoring should be implemented in clinical trials assessing retinoids.

Despite the fact that palovarotene 5.0 mg was associated with a non-statistically significant reduction from baseline in OC and OC cartilage volume, given the adverse bone safety events observed with palovarotene^18^, no further studies of palovarotene in MHE will be conducted by the Sponsor. Future study directions should focus on improving the molecular understanding of MHE, which may reveal additional treatment targets. Current potential treatment targets include bone morphogenic protein (BMP) and Hedgehog signaling pathways, and the enzyme heparinase^31^.

In conclusion, the results of MO-Ped indicated a non-favorable benefit-risk profile for palovarotene in MHE, with no significant differences in the annualized rate of new OCs, or in change from baseline in volume of OCs or OC cartilage, observed between treatment groups. Interpretation of results was limited by the reduced treatment duration and the smaller than expected treatment cohort following early trial termination. Despite these limitations preventing definitive conclusions regarding efficacy, these data provide pertinent insight into understanding of the natural history of MHE in pediatric patients and the data gathered may inform future trials or therapeutic approaches in MHE.

## Supplementary Information

Below is the link to the electronic supplementary material.

Supplementary Material 1

## Data Availability

Qualified researchers with a valid research question may request anonymised patient-level data by contacting an Ipsen representative. Further information on Ipsen’s Data Sharing policy is available here (Clinical Data Transparency - Global).
