# Supplementary material for: Palovarotene for patients with multiple hereditary exostosis: results of MO-Ped, a terminated, randomized, placebo-controlled, double-blind phase 2 trial
Source: Sci Rep. 2025 Nov 4;15:38563. doi: 10.1038/s41598-025-22554-6 (PMC12586466; doi:10.1038/s41598-025-22554-6)
Supplement: Supplementary file 1 — Supplementary Material 1 [file 41598_2025_22554_MOESM1_ESM.docx]

**Palovarotene for Patients with Multiple Hereditary Exostosis: Results of MO-Ped, a Terminated, Randomized, Placebo‑Controlled, Double-Blind Phase 2 Trial**

Luca Sangiorgi, MD (ORCID: 0000-0003-3658-1209),^1^ Ernest U. Conrad, MD (ORCID: 0000-0002-0485-907X),^2^ Fei Shih, MD, PhD (ORCID: 0009-0006-5821-1267),^3^ Andrew Strahs, PhD,^3^ David S. Feldman, MD (ORCID: 0000-0001-9190-6083)^4^

^1^Medical Genetics and Skeletal Rare Diseases, IRCCS Istituto Ortopedico Rizzoli, Bologna, Italy; ^2^Department of Orthopedic Surgery, University of Texas Health Science Center, Houston, TX, U.S.; ^3^Ipsen, Cambridge, MA, U.S.; ^4^Paley Advanced Limb Lengthening Institute, St. Mary's Hospital, West Palm Beach, FL, U.S.

**Corresponding author:** Fei Shih ([fei.shih@ipsen.com](mailto:fei.shih@ipsen.com))

***Calcified Tissue International***

**SUPPLEMENTARY MATERIAL**

PLAIN LANGUAGE SUMMARY

Multiple hereditary exostosis (MHE) is a rare condition in which people develop bone tumors called osteochondromas. Growth of osteochondromas occurs during childhood, which can cause difficulty walking and pain. Osteochondromas can sometimes be removed by surgery, but patients may need many operations. A medicine called palovarotene was studied in this clinical trial to understand if it can prevent or reduce growth of osteochondromas and is tolerated in children with MHE.

The clinical trial included 194 children with MHE aged between 2 and 14 years. Their bones were scanned yearly to check for new and growing osteochondromas. In December 2019, the trial was stopped early because of concerns that palovarotene may cause early closure of growth plates, which are important for growth during childhood.

When the trial was stopped, 30 children had already received treatment for at least 1 year, and had their 1-year bone scans taken. 12 of these children received a low dose (2.5 mg) of palovarotene, 10 had a higher dose (5.0 mg) of palovarotene, and 8 received a tablet with no medicine (placebo). Results showed that palovarotene did not change the number of new osteochondromas or the size of osteochondromas. Most side effects of palovarotene treatment were mild, such as dry and itchy skin. There was no early growth plate closure in children treated with palovarotene, but bone growth tended to be slower compared with those who received placebo. Overall, palovarotene did not appear to be a good treatment option for children with MHE.

SUPPLEMENTARY METHODS

***Trial Design***

Symptomatic MHE was defined by the presence of at least one of the following symptoms: ≥5 clinically evident osteochondromas (OCs) and the presence of a new or enlarging OC in the preceding 12 months; ≥5 clinically evident OCs and the presence of a painful OC; the presence of a skeletal deformity; the presence of a joint limitation; or prior surgery for an MHE-related complication. Patients must have been able to undergo whole-body magnetic resonance imaging (MRI) with or without sedation/general anesthesia. Bone age at screening was required to be ≤14 years, as per the Greulich-Pyle method, and female patients were required to be premenarchal. All patients were required to provide written, signed, and dated informed patient/parent consent, and age-appropriate assent where required by local guidelines. Patients’ race and ethnicity were self-reported.

Patients with amylase or lipase >2 times the upper limit of normal (ULN), aminotransferase or alanine aminotransferase >2.5 times ULN, a history of chronic pancreatitis or other syndromic conditions (such as Langer-Giedion or Potocki-Shaffer syndrome), concomitant medications which affect cytochrome P450 3A4 activity, or body weight <10 kg were excluded.

Patients were enrolled at 29 trial sites in 11 countries: Australia (n=1), Belgium (n=1), Canada (n=3), France (n=2), Japan (n=2), Netherlands (n=1), Portugal (n=1), Spain (n=1), Turkey (n=2), U.K. (n=3), and U.S. (n=12).

Palovarotene was administered orally with food at approximately the same time each day and doses were weight-adjusted for patients <60 kg (**Supplementary Table 1**).

***Whole-Body MRI Acquisition and Analysis***

Whole-body MRI scans, involving coronal acquisitions from the thoracic girdle to the ankles, sagittal acquisitions of the upper extremities and lower extremities, and transversal acquisitions of the thoracic and pelvic girdles, were performed at Baseline, Month 12, and Month 24 to assess these endpoints. All images were interpreted by a central imaging laboratory blinded to the trial using standardized procedures. Briefly, the initial evaluation involved assessing each bone to identify OCs. Subsequently, each OC was outlined slice-by-slice and regions of interest (ROIs) for the osseous and cartilage components were recorded. Quantitative assessments performed on these were similar to response evaluations in solid tumors (RECIST guidelines);^1^ minimal size for measurability was 10 mm in the longest diameter and a putative OC must have been documented on ≥2 consecutive slices to ensure reliability. All relevant ROIs for each OC were integrated to calculate overall OC volume.

***Definitions of Adverse Events***

Adverse events (AEs) that developed, worsened, or became serious during the treatment period were defined as treatment-emergent AEs (TEAEs) and standardized definitions were used when determining the relationship of study treatment to an AE. AEs that led to life-threatening situations, inpatient hospitalization, or persistent/significant disability were defined as serious AEs.

***Additional Safety Outcomes***

Bone safety evaluations included: growth assessment (evaluated by linear height and knee height); growth plate assessment (evaluated by radiographs); bone mineral content (BMC) and areal bone mineral density (aBMD) at the lumbar spine, hip and the mid-third radius (evaluated by dual energy x-ray absorptiometry [DXA]); incidence of possible vertebral compression fractures (VCFs; evaluated by MRI/DXA); and osteonecrosis of the hips, shoulders, and knees (evaluated by MRI). Other safety evaluations included: electrocardiograms (ECGs), blood/urine samples for clinical laboratory tests, vital signs, body weight, and the assessment of suicidal ideation/behavior (using the Columbia Suicide Severity Rating Scale).^2^ Concomitant medications were assessed at each site and remote visit.

***Statistical Analyses***

The annualized rates of new OCs, new or worsening deformities, and MHE-related surgeries were estimated using negative binomial regression models, offset by log-transformed follow-up time (years). Changes from Baseline in surgery-adjusted volume of OCs and OC cartilage were summarized using descriptive statistics; surgery-adjustment was conducted to ensure that volume losses due to surgical removal of symptomatic OCs did not contribute towards the endpoints. Surgery-adjusted changes in volume were determined by adding Baseline volume for OCs on which surgery was performed between Baseline and Month 12 to the reported Month 12 OC volume. The mean difference in change from Baseline volumes was estimated using generalized estimating equation models. Covariates included in the models were treatment, Baseline age, sex, and *EXT1/EXT2* variant status. As siblings were enrolled in the trial, repeated measures with an unstructured covariance matrix were used to address potential correlation between family members. The proportion of patients with no new OCs was reported using descriptive statistics and the exact confidence interval, derived using the Clopper-Pearson method.

For all efficacy endpoints, point estimates for each treatment group were compared with all other treatment groups (i.e., placebo versus palovarotene 2.5 mg, placebo versus palovarotene 5.0 mg, and palovarotene 2.5 mg versus palovarotene 5.0 mg). The p‑values presented were not adjusted for multiple testing due to the small sample size.

SUPPLEMENTARY TABLES

**Supplementary Table 1** Weight-adjusted palovarotene doses

| **Weight category** | **Palovarotene 2.5 mg equivalent** | **Palovarotene 5.0 mg equivalent** |
| --- | --- | --- |
| 10 to <20 kg | 1.0 mg | 2.5 mg |
| 20 to <40 kg | 1.5 mg | 3.0 mg |
| 40 to <60 kg | 2.0 mg | 4.0 mg |
| ≥60 kg | 2.5 mg | 5.0 mg |

**Supplementary Table 2** List of Independent Ethics Committees or Institutional Review Boards

| Country/ Site Number | Ethics Committee |
| --- | --- |
| USA 1001 | University of Texas Health Science Center at Houston Institutional Review Board  6410 Fannin Street, Suite 1100  Houston, TX 77030 |
| USA 1002 | Mayo Clinic Institutional Review Board  200 First Street Southwest  201 Building, Room 4-60  Rochester, MN 55905 |
| USA 1003 | MetroWest Medical Center Institutional Review Board  115 Lincoln Street,  Framingham, Mass.1702 |
| USA 1004 | UCSF Human Research Protection Program  3333 California Street, Suite 315  San Francisco, 94118 CA |
| USA 1005 | Boston Children's Hospital IRB  300 Longwood Avenue  Mailstop: BCH 3164  Boston, 2115 Mass. |
| USA 1006 | Western Institutional Review Board  1019 39th Avenue Southeast, Suite120  Puyallup, 98374-2115 WA |
| USA 1007 | Children's Hospital Los Angeles Institutional Review Board  4650 Sunset Boulevard,  Mail Stop 23  Los Angeles, 90027 CA |
| USA 1008 | The Children's Hospital of Philadelphia (CHOP) IRB  2716 South Street, 4th Floor  Philadelphia, 19146 PSU |
| USA 1009 | Western Institutional Review Board  1019 39th Avenue Southeast, Suite  120,  Puyallup 98374-2115 WA |
| USA 1010 | Western Institutional Review Board  1019 39th Avenue Southeast, Suite  120,  Puyallup 98374-2115 WA |
| USA 1011 | Johns Hopkins Medicine Office of Human Subjects Research Institutional Review Board  1620 Mcelderry Street, Reed Hall,  B130  Baltimore, 21205-1911 Md. |
| USA 1012 | Western Institutional Review Board  1019 39th Avenue Southeast, Suite  120,  Puyallup 98374-2115 WA |
| USA 1013 | Western Institutional Review Board  1019 39th Avenue Southeast, Suite 120,  Puyallup 98374-2115 WA |
| Canada 1101 | SickKids REB  555 University Avenue  Toronto, M5G 1X8 Ontario |
| Canada 1102 | Comité D'Ethique du Centre Hospitalier  Universitaire Ste Justine  3175 Cote Sainte-Catherine, Montreal  H3T 1C5, Quebec |
| Canada 1103 | McGill University Faculty of Medicine Institutional Review Board (IRB)  3655 Promenade Sir William Osler,  McIntyre Medical Building, Suite 633,  Montreal H3G 1Y6 Quebec |
| Italy 2001 | Comitato Etico Indipendente di Area Vasta Emilia Centro (CE-AVEC) della Regione Emilia-Romagna  Via Albertoni, 15 40138 Bologna (BO),  Italy |
| Spain 2201 | CEIC Hospital Universitario La Paz  Paseo de la Castellana, 261,  Hospital General - Comité Ético de  Investigación Clínica, Planta 8, Madrid  28046 Spain |
| France 2401/2402 | CPP Sud-Est V  6 Boulevard de la Chantourne,  Couloir Sce de Médecine Légale / Pôle des sourds,  Rez de Chausée Haut – Hall Vercors,  La Tronche 38700 France |
| UK 2501/2502/2503 | Health Research Authority (HRA)  80 London Road,  London SE1 6LH, UK |
| UK 2501/2502 | NRES Committee North West - Greater Manchester South  4 Minshull Street,  Manchester M1 3DZ, UK |
| Belgium 2701 | UZ Antwerpen Wilrijkstraat 10,  Edegem, Antwerpen 2650, Belgium |
| Turkey 2801/2802 | Bezmiâlem Vakıf Üniversitesi Klinik  Araştırmalar Etik Kurulu  Adnan Menderes Bulvarı Vatan  Caddesi, Istanbul 34093, Turkey |
| Portugal 2901 | Comissão de Ética para a Investigação Clínica – CEIC  Avenida do Brasil, 53  Pavilhão 17-A, Lisoba 1749-004,  Lisoba Portugal |
| Netherlands 3001 | METC Brabant  Hilvarenbeekseweg 60, Tilburg, 5022  GC Noord-Brabant, Netherlands |
| Australia 5001 | Sydney Childrens Hospital Network HREC  High Street, Randwick, 2031 NSW,  Australia |
| Japan 5101 | Osaka University Hospital Institutional Review Board  2-2 Yamadaoka, Department of Medical innovation,  4th floor of The Center of Medical Innovation and Translational Research,  Suita, 565-0871 Ôsaka, Japan |
| Japan 5102 | Nagoya University Hospital Institutional Review Board,  65 Tshutumai-cho, Showa-ku, Nagoya,  466-8560 Aichi, Japan |

**Supplementary Table 3** Schedule of assessments planned in MO‑Ped

|  | **Screening** | **Baseline/  treatment** | **Treatment** | | | | **EOT** | **Safety  follow-up** | |
| --- | --- | --- | --- | --- | --- | --- | --- | --- | --- |
|  | **−35 Days** | **Study Day 1** | **Month 1  ±7 days** | **Months 3, 9, 15, 21 ±7 days^a^** | **Months**  **6, 18 ±7 days** | **Month 12 ±7 days** | **Month 24 ±7 days** | **4 weeks after EOT** | **6 months after EOT^b^** |
| **Assessment/Procedure** | **Site** | **Site** | **Remote** | **Remote** | **Site** | **Site** | **Site** | **Remote** | **Site** |
| Informed consent/assent | X |  |  |  |  |  |  |  |  |
| Assessment of inclusion/exclusion criteria | X | X |  |  |  |  |  |  |  |
| MRI sedation screening | X |  |  |  |  |  |  |  |  |
| Knee and hand/wrist radiograph for assessment of growth plate^c^ | X |  |  |  | X | X | X |  | X |
| Genotyping | X |  |  |  |  |  |  |  |  |
| Linear growth assessment (by stadiometry) and bilateral knee height (by caliper)^d^ |  | X |  |  | X | X | X |  | X |
| Medical history | X |  |  |  |  |  |  |  |  |
| Physical examination | X | X |  |  | X | X | X |  | X |
| Hearing and visual acuity tests |  | X |  |  |  | X | X |  |  |
| Tanner staging | X |  |  |  |  | X | X |  |  |
| C-SSRS^e^ | X |  |  | X | X | X | X |  |  |
| PedsQL^f^, PROMIS^g^, FPS-R^h^ |  | X |  |  | X | X | X |  |  |
| Joint range of motion |  | X |  |  | X | X | X |  |  |
| Body weight | X | X |  | X | X | X | X |  |  |
| Vital signs | X | X |  | X | X | X | X | X | X |
| Electrocardiogram | X | X |  |  |  | X | X |  |  |
| Hematology | X | X |  | X^i^ | X | X | X | X | X |
| Biochemistry (includes lipids) | X | X |  | X^i^ | X | X | X | X | X |
| Parathyroid hormone |  | X |  | X | | | |  |  |
| Pharmacokinetics |  |  | X |  |  |  |  |  |  |
| Urinalysis | X | X |  | X^i^ | X | X | X | X | X |
| Blood pregnancy test | X |  |  | X | X | X | X |  |  |
| Urine pregnancy test |  | X | Every month except at visits when blood samples were obtained | | | | | X |  |
| Whole-body MRI |  | X |  |  |  | X | X |  |  |
| Radiographs of upper/lower limbs  (weight bearing) |  | X |  |  |  | X | X |  |  |
| DXA |  | X |  |  | X | X | X |  | X |
| Randomization by IWRS |  | X |  |  |  |  |  |  |  |
| Study treatment dispensing |  | X |  |  | X | X |  |  |  |
| Study treatment accounting |  |  |  |  | X | X | X |  |  |
| Treatment |  | Treatment (once daily) | | | | | |  |  |
| Palatability of sprinkled product |  | X | X |  |  |  |  |  |  |
| Prior/concomitant medications | At every patient contact | | | | | | | | |
| Inquiry of MHE-related surgeries | At every patient contact | | | | | | | | |
| Adverse events | At every patient contact | | | | | | | | |

^a^Visit times followed the original scheduled visit whenever possible. ^b^If the safety laboratory results were normal at the 4-week safety follow-up visit, they did not need to be repeated at the 6‑month safety follow‑up visit. ^c^Hand/wrist and knee radiographs were obtained at screening and every 6 months. ^d^Measurements of linear growth and bilateral knee height were performed in triplicate; patients with no observed growth from the preceding height measurement were reassessed after 3 months to confirm if growth was halted. ^e^Assessed in patients aged ≥8 years. ^f^For PedsQL assessments, the Parent Report for Toddlers was used for patients aged 2–4 years, Parent Report for Young Children for patients aged 5–7 years, Child Report (self-reporting) for patients aged 8–12 years, and the Teen Report (self-reporting) for patients aged ≥13 years. ^g^PROMIS assessments used the parent proxy short form for patients aged 2–7 years and the pediatric short form (self-reporting) for patients aged ≥8 years. ^h^Assessed in patients aged ≥4 years. ^i^Hematology, biochemistry, and urinalysis at Months 15 and 21 were waived if no laboratory abnormalities were observed during the first 12 months of study treatment. C-SSRS, Columbia-Suicide Severity Rating Scale; DXA, dual x-ray absorptiometry; EOT, end of treatment; FPS-R, Faces Pain Scale – Revised; IWRS, Interactive Web Response System; MHE, multiple hereditary exostosis; MRI, magnetic resonance imaging; PedsQL, Pediatric Quality of Life Inventory; PROMIS, Patient‑Reported Outcomes Measurement Information System.

**Supplementary Table 4** Baseline demographics and characteristics for patients in the Safety Set^a^

|  | **Placebo (N=62)** | **Palovarotene 2.5 mg (N=66)** | **Palovarotene 5.0 mg (N=65)** | **All palovarotene-treated patients (N=131)** | **All patients (N=193)** |
| --- | --- | --- | --- | --- | --- |
| **Sex, n (%)** |  |  |  |  |  |
| Male | 39 (62.9) | 40 (60.6) | 38 (58.5) | 78 (59.5) | 117 (60.6) |
| **Race, n (%)^b^** | | | | | |
| White  Asian  Black or African American  American Indian or Alaska Native  Multiple  Other  Missing | 48 (77.4)  5 (8.1)  0  0  6 (9.7)  0  3 (4.8) | 50 (75.8)  3 (4.5)  2 (3.0)  1 (1.5)  7 (10.6)  0  3 (4.5) | 52 (80.0)  3 (4.6)  2 (3.1)  0  5 (7.7)  1 (1.5)  2 (3.1) | 102 (77.9)  6 (4.6)  4 (3.1)  1 (0.8)  12 (9.2)  1 (0.8)  5 (3.8) | 150 (77.7)  11 (5.7)  4 (2.1)  1 (0.5)  18 (9.3)  1 (0.5)  8 (4.1) |
| **Ethnicity, n (%)^b^** | | | | | |
| Hispanic or Latino  Missing | 6 (9.7)  0 | 7 (10.6)  1 (1.5) | 7 (10.8)  1 (1.5) | 14 (10.7)  2 (1.5) | 20 (10.4)  2 (1.0) |
| **Age, years** |  |  |  |  |  |
| Mean (SD) | 7.9 (2.5) | 7.8 (3.1) | 7.4 (3.1) | 7.6 (3.1) | 7.7 (2.9) |
| Median (min, max) | 8.0 (3, 12) | 8.0 (2, 13) | 8.0 (2, 14) | 8.0 (2, 14) | 8.0 (2, 14) |
| **Age group, n (%)** |  |  |  |  |  |
| 2–5 years | 13 (21.0) | 17 (25.8) | 19 (29.2) | 36 (27.5) | 49 (25.4) |
| 6–10 years | 38 (61.3) | 33 (50.0) | 35 (53.8) | 68 (51.9) | 106 (54.9) |
| 11–14 years | 11 (17.7) | 16 (24.2) | 11 (16.9) | 27 (20.6) | 38 (19.7) |
| **Age at diagnosis, years** |  |  |  |  |  |
| Mean (SD) | 2.9 (2.15) | 3.0 (2.09) | 3.0 (2.24) | 3.0 (2.16) | 3.0 (2.15) |
| Median (min, max) | 2.8 (0, 10) | 2.2 (0, 9) | 3.0 (0, 11) | 2.4 (0, 11) | 2.5 (0, 11) |
| **Time since diagnosis, years** |  |  |  |  |  |
| Mean (SD) | 5.6 (3.15) | 5.3 (3.07) | 5.0 (3.23) | 5.2 (3.14) | 5.3 (3.14) |
| Median (min, max) | 5.5 (1, 12) | 5.1 (1, 12) | 4.7 (0, 14) | 5.0 (0, 14) | 5.0 (0, 14) |
| **MHE surgeries performed, n (%)** | | | | | |
| Yes | 32 (51.6) | 39 (59.1) | 32 (49.2) | 71 (54.2) | 103 (53.4) |
| **Pain, n (%)** | | | | | |
| Yes | 24 (38.7) | 24 (36.4) | 25 (38.5) | 49 (37.4) | 73 (37.8) |
| **Joint deformity, gait or posture disturbance, n (%)** | | | | | |
| Yes | 28 (45.2) | 24 (36.4) | 19 (29.2) | 43 (32.8) | 71 (36.8) |
| **Has other family members with MHE, n^c^** | 62 | 66 | 64 | 130 | 192 |
| Mother, n (%)^d^ | 18 (29.0) | 19 (28.8) | 22 (33.8) | 41 (31.3) | 59 (30.6) |
| Father, n (%)^d^ | 23 (37.1) | 14 (21.2) | 18 (27.7) | 32 (24.4) | 55 (28.5) |
| Siblings, n (%)^d^ | 23 (37.1) | 20 (30.3) | 26 (40.0) | 46 (35.1) | 69 (35.8) |
| Other family, n (%)^d^ | 17 (27.4) | 23 (34.8) | 18 (27.7) | 41 (31.3) | 58 (30.1) |
| None, n (%)^d^ | 18 (29.0) | 30 (45.5) | 23 (35.4) | 53 (40.5) | 71 (36.8) |
| **MHE-associated genetic variant, n (%)** | | | | | |
| *EXT1* | 49 (79.0) | 51 (77.3) | 50 (76.9) | 101 (77.1) | 150 (77.7) |
| *EXT2* | 13 (21.0) | 15 (22.7) | 15 (23.1) | 30 (22.9) | 43 (22.3) |
| **MHE-associated mutation type, n (%)** | | | | | |
| Missense | 8 (12.9) | 8 (12.1) | 11 (16.9) | 19 (14.5) | 27 (14.0) |
| Nonsense | 19 (30.6) | 16 (24.2) | 21 (32.3) | 37 (28.2) | 56 (29.0) |
| Silent | 0 | 0 | 1 (1.5) | 1 (0.8) | 1 (0.5) |
| Donor-acceptor splice site | 7 (11.3) | 10 (15.2) | 8 (12.3) | 18 (13.7) | 25 (13.0) |
| Copy number variants (exon-level deletion/duplications) and whole gene deletion/duplications | 8 (12.9) | 8 (12.1) | 7 (10.8) | 15 (11.5) | 23 (11.9) |
| Intronic | 1 (1.6) | 0 | 4 (6.2) | 4 (3.1) | 5 (2.6) |
| Frame shift | 14 (22.6) | 19 (28.8) | 10 (15.4) | 29 (22.1) | 43 (22.3) |
| Stop-loss | 1 (1.6) | 2 (3.0) | 1 (1.5) | 3 (2.3) | 4 (2.1) |
| In-frame deletion or duplication | 4 (6.5) | 2 (3.0) | 2 (3.1) | 4 (3.1) | 8 (4.1) |
| Other | 0 | 1 (1.5) | 0 | 1 (0.8) | 1 (0.5) |

^a^All patients who received at least one dose of study treatment. ^b^Self-reported. ^c^Number of patients assessed. ^d^Percentages are given relative to the total population, N. *EXT1*, exostosin 1; *EXT2,* exostosin 2; max, maximum; MHE, multiple hereditary exostosis; min, minimum; SD, standard deviation.

**Supplementary Table 5** Baseline demographics and characteristics for patients who completed Month 12 efficacy imaging

|  | **Placebo (N=16)** | **Palovarotene 2.5 mg (N=17)** | **Palovarotene 5.0 mg (N=23)** | **All palovarotene-treated patients (N=40)** | **All patients (N=56)** |
| --- | --- | --- | --- | --- | --- |
| **Sex, n (%)** |  |  |  |  |  |
| Male | 11 (68.8) | 11 (64.7) | 13 (56.5) | 24 (60.0) | 35 (62.5) |
| **Race, n (%)** | | | | | |
| White  Asian  Multiple  Missing | 13 (81.3)  1 (6.3)  1 (6.3)  1 (6.3) | 14 (82.4)  2 (11.8)  1 (5.9)  0 | 19 (82.6)  1 (4.3)  3 (13.0)  0 | 33 (82.5)  3 (7.5)  4 (10.0)  0 | 46 (82.1)  4 (7.1)  5 (8.9)  1 (1.8) |
| **Ethnicity, n (%)** | | | | | |
| Hispanic or Latino | 1 (6.3) | 2 (11.8) | 2 (8.7) | 4 (10.0) | 5 (8.9) |
| **Age, years** |  |  |  |  |  |
| Mean (SD) | 7.8 (3.2) | 7.7 (3.9) | 7.8 (2.8) | 7.8 (3.2) | 7.8 (3.2) |
| Median (min, max) | 7.5 (3, 12) | 8.0 (2, 12) | 8.0 (3, 12) | 8.0 (2, 12) | 8.0 (2, 12) |
| **Age group, n (%)** |  |  |  |  |  |
| 2–5 years | 6 (37.5) | 5 (29.4) | 6 (26.1) | 11 (27.5) | 17 (30.4) |
| 6–10 years | 5 (31.3) | 5 (29.4) | 12 (52.2) | 17 (42.5) | 22 (39.3) |
| 11–14 years | 5 (31.3) | 7 (41.2) | 5 (21.7) | 12 (30.0) | 17 (30.4) |
| **MHE-associated genetic variant, n (%)** | | | | | |
| *EXT1* | 12 (75.0) | 12 (70.6) | 16 (69.6) | 28 (70.0) | 40 (71.4) |
| *EXT2* | 4 (25.0) | 5 (29.4) | 7 (30.4) | 12 (30.0) | 16 (28.6) |
| **MHE-associated mutation type, n (%)** | | | | | |
| Missense | 4 (25.0) | 1 (5.9) | 5 (21.7) | 6 (15.0) | 10 (17.9) |
| Nonsense | 2 (12.5) | 4 (23.5) | 8 (34.8) | 12 (30.0) | 14 (25.0) |
| Silent | 0 | 0 | 1 (4.3) | 1 (2.5) | 1 (1.8) |
| Donor-acceptor splice site | 2 (12.5) | 3 (17.6) | 1 (4.3) | 4 (10.0) | 6 (10.7) |
| Copy number variants (exon-level deletions/duplications) and whole gene deletions/duplications | 2 (12.5) | 2 (11.8) | 2 (8.7) | 4 (10.0) | 6 (10.7) |
| Intronic | 0 | 0 | 1 (4.3) | 1 (2.5) | 1 (1.8) |
| Frame shift | 3 (18.8) | 7 (41.2) | 4 (17.4) | 11 (27.5) | 14 (25.0) |
| In-frame deletion or duplication | 3 (18.8) | 0 | 1 (4.3) | 1 (2.5) | 4 (7.1) |

*EXT1*, exostosin 1; *EXT2,* exostosin 2; max, maximum; MHE, multiple hereditary exostosis; min, minimum; SD, standard deviation.

SUPPLEMENTARY FIGURES

Supplementary Fig 1 Planned MO-Ped trial design


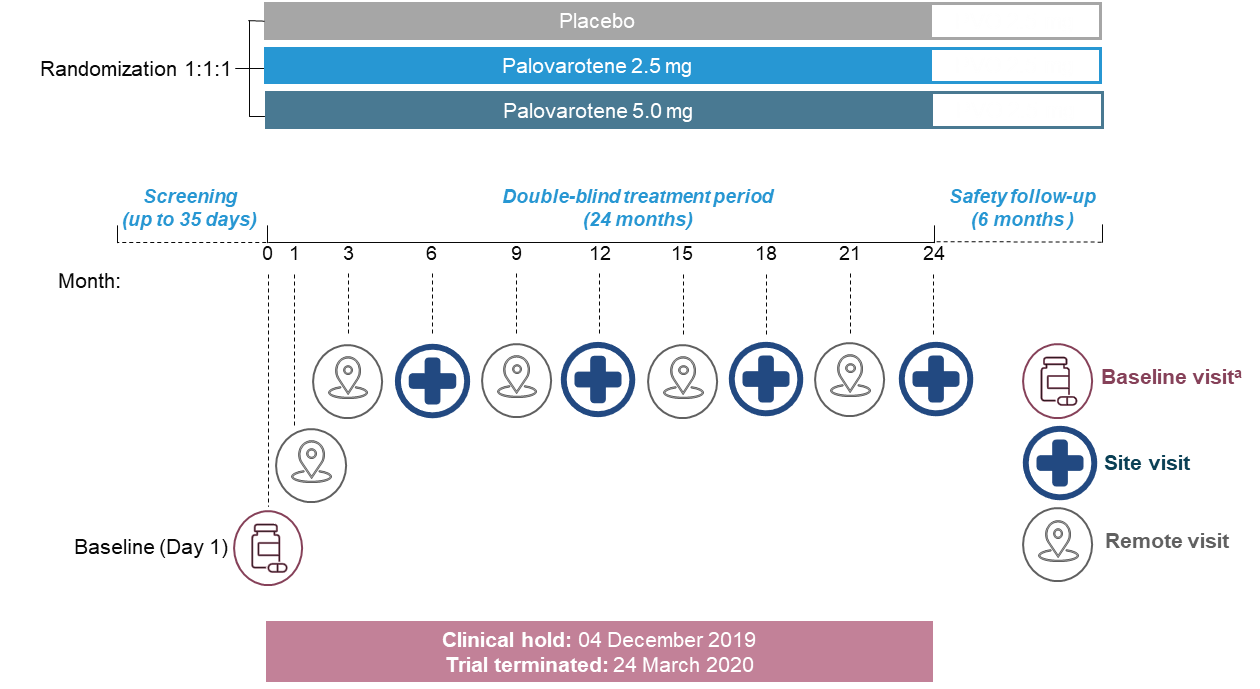


^a^Patients were examined by whole-body MRI and radiographs of the upper and lower limbs to determine the number, size, and location of OCs, joint deformities, and other skeletal abnormalities. MRI, magnetic resonance imaging; OC, osteochondroma.

Supplementary Fig 2 Efficacy results for patients who completed Month 12 efficacy imaging

1. Annualized rate of new OCs at Month 12^a^


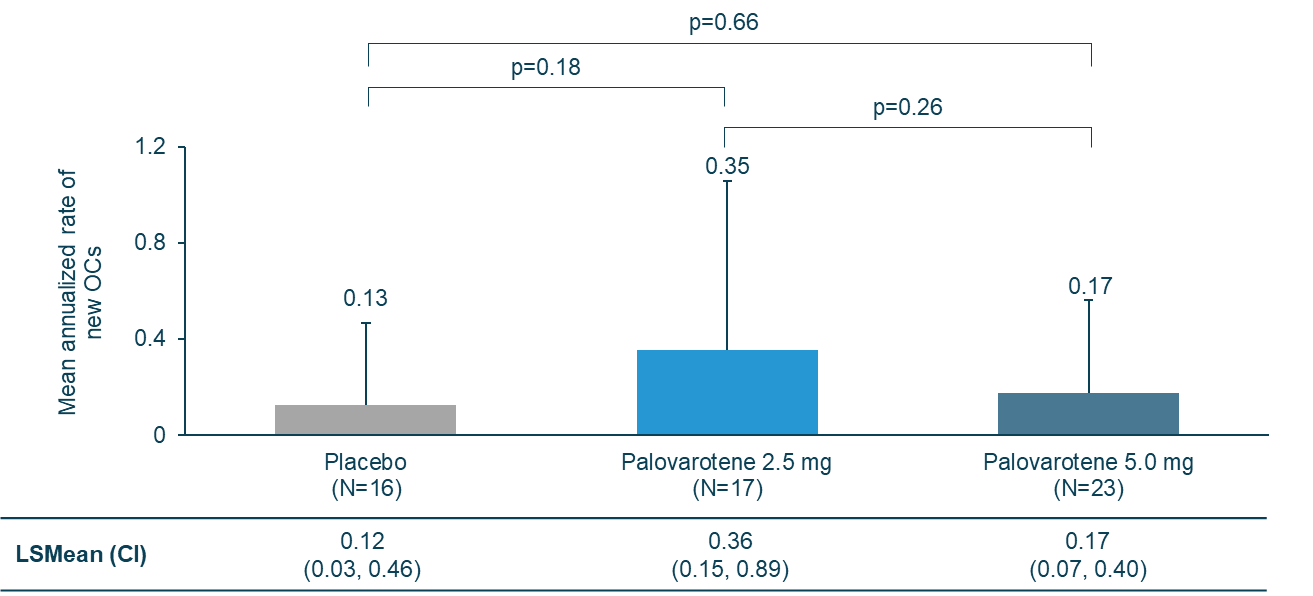


1. Mean change from Baseline in surgery-adjusted volume of OCs at Month 12


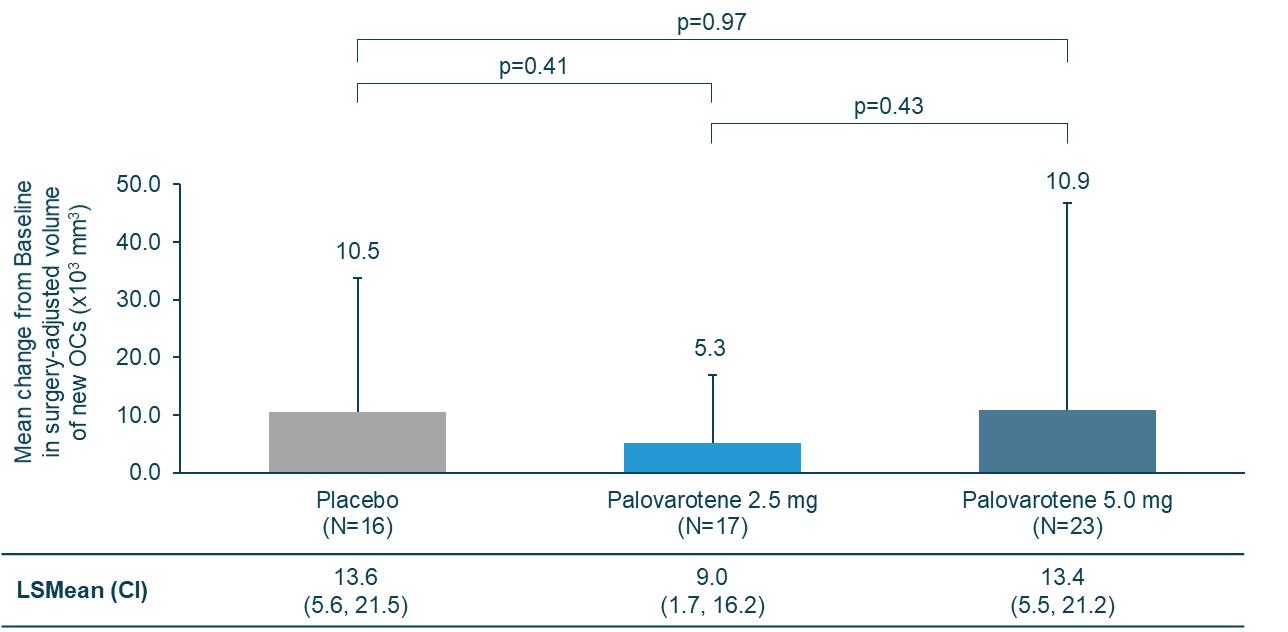


1. **
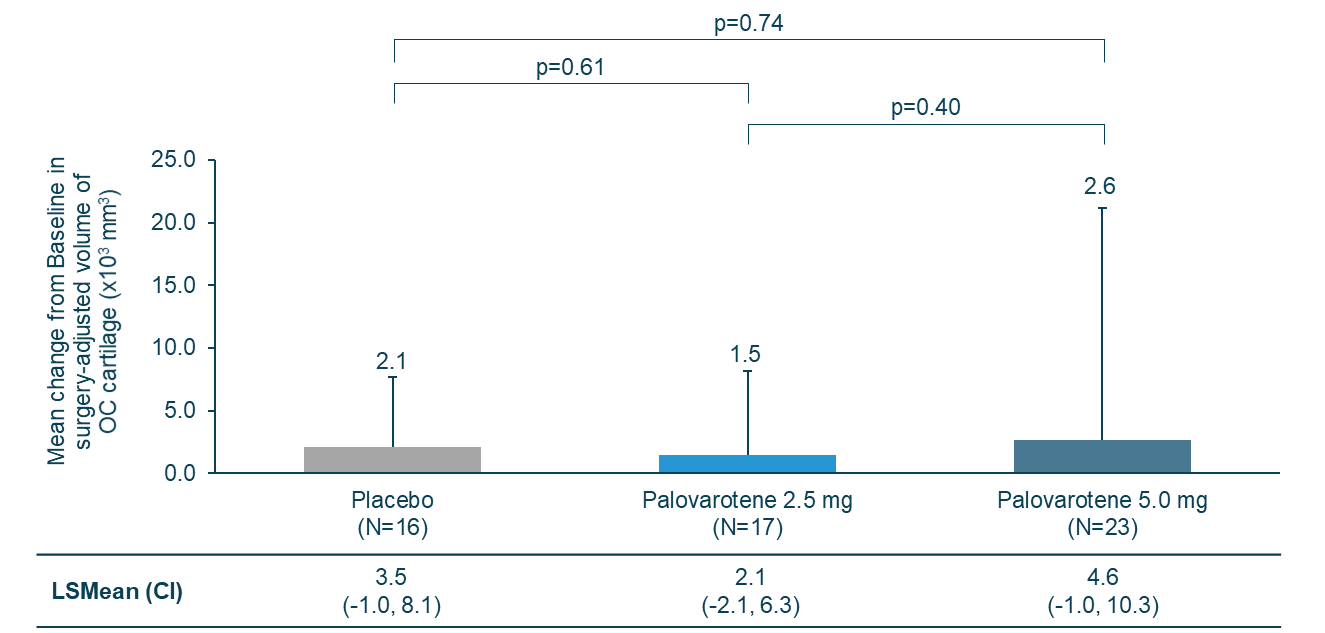
** Mean change from Baseline in surgery-adjusted volume of OC cartilage at Month 12

^a^Determined by the number of new OCs identified by MRI; therefore, surgically-removed OCs were not included. Error bars show SDs of the mean. CI, confidence interval; LSMean, least squares mean; MRI, magnetic resonance imaging; OC, osteochondroma; SD, standard deviation.

Supplementary Fig 3 Additional efficacy results

1. Proportion of patients with no new OCs at Month 12 in those patients at risk of developing new OCs^a^


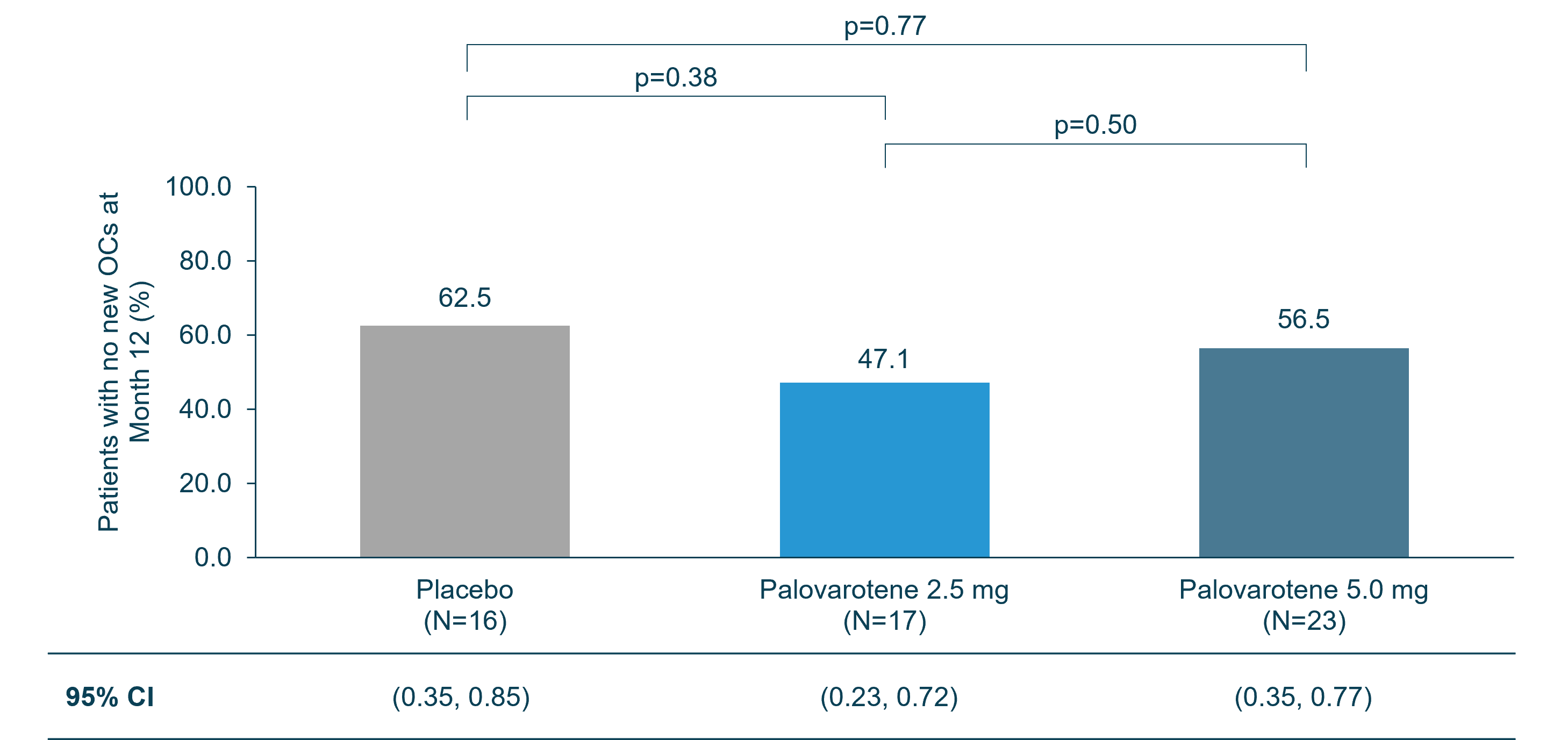


1. Annualized rate of new or worsening skeletal deformities for patients who completed Month 12 efficacy imaging

**
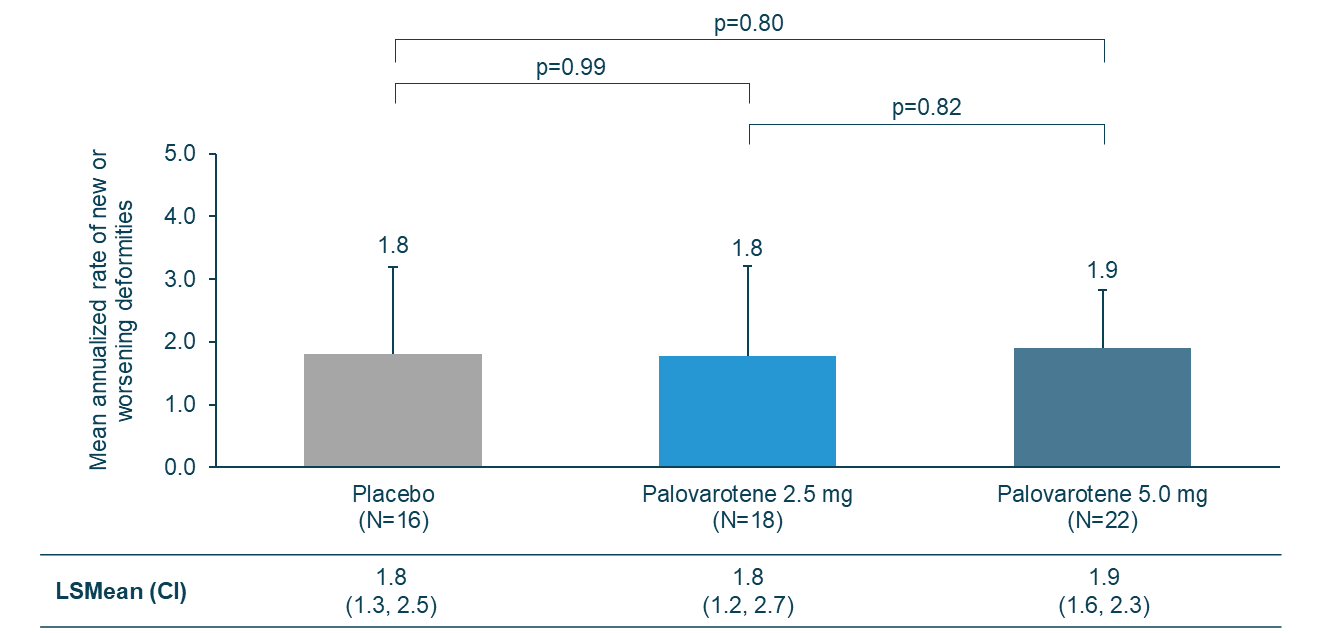
**

1. Annualized rate of MHE-related surgeries for patients who completed Month 12 efficacy imaging


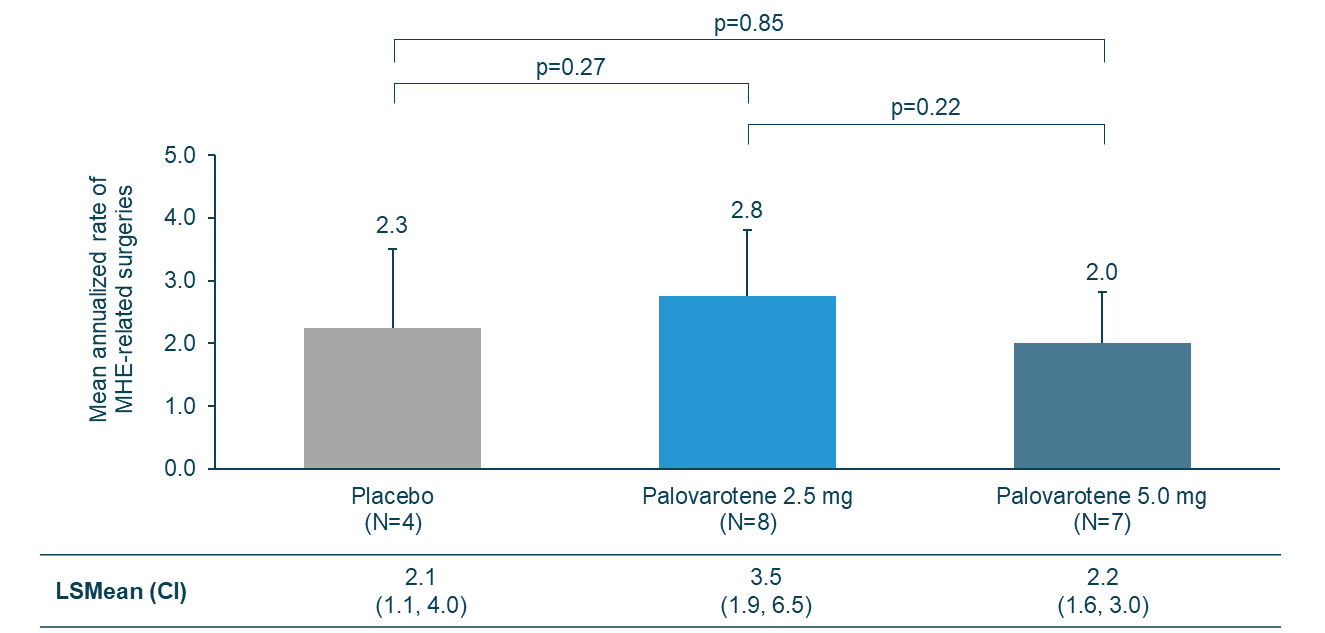


^a^OCs that were surgically removed over the course of the study were considered in the analysis. Error bars show SDs of the mean. (A) Patients with new OCs not identified by MRI due to surgical resection during the treatment period were categorized as having new OCs for this analysis. (B) The annualized rate for number of new or worsening deformities was estimated using a negative binomial regression model, offset by log-transformed follow-up time (years) to obtain annualized rate. (C) The annualized rate for number of MHE-related surgeries was estimated using a Poisson regression model, offset by log-transformed follow-up time (years) to obtain annualized rate. MHE-related surgeries included any procedure indicated for the treatment of MHE, which may have included excisions to eliminate symptomatic OCs or procedures to correct deformities and/or functional limitations. Patients with planned surgeries within 6 months of enrolment to remove symptomatic OCs or to correct deformities present at Baseline, and/or had surgical procedures that were a continuation of a previous procedure were excluded. CI: confidence interval; LSMean: least squares mean; MHE, multiple hereditary exostosis; MRI, magnetic resonance imaging; OC: osteochondroma; SD: standard deviation.

1. Eisenhauer EA, Therasse P, Bogaerts J, et al. New response evaluation criteria in solid tumours: revised RECIST guideline (version 1.1). Eur J Cancer 2009;45:228-247.

2. Posner K, Brown GK, Stanley B, et al. The Columbia-Suicide Severity Rating Scale: initial validity and internal consistency findings from three multisite studies with adolescents and adults. Am J Psychiatry 2011;168:1266-1277.
